# Continuity of care: what matters to women when they are referred from primary to secondary care during labour? a qualitative interview study in the Netherlands

**DOI:** 10.1186/1471-2393-14-103

**Published:** 2014-03-17

**Authors:** Ank de Jonge, Rosan Stuijt, Iva Eijke, Marjan J Westerman

**Affiliations:** 1 Department of Midwifery Science, AVAG/EMGO Institute of Health and Care Research, VU University Medical Center, Van der Boechorststraat 7, 1081 BT Amsterdam, The Netherlands; 2 Department of Methodology and Statistics, Institute of Health Sciences and the EMGO Institute of Health and Care Research, VU University Amsterdam, De Boelelaan 1085, 1081 HV Amsterdam, The Netherlands

**Keywords:** Continuity of patient care, Home childbirth, Birth experience, Intrapartum care, Primary care

## Abstract

**Background:**

Continuity of care during labour is important for women. Women with an intrapartum referral from primary to secondary care look back more negatively on their birh experience compared to those who are not referred. It is not clear which aspects of care contribute to this negative birth experience. This study aimed to explore in-depth the experiences of women who were referred during labour from primary to secondary care with regard to the different aspects of continuity of care.

**Methods:**

A qualitative interview study was conducted in the Netherlands among women who were in primary care at the onset of labour and were referred to secondary care before the baby was born. Through purposive sampling 27 women were selected. Of these, nine women planned their birth at home, two in an alongside midwifery unit and 16 in hospital. Thematic analysis was used.

**Results:**

Continuity of care was a very important issue for women because it contributed to their feeling of safety during labour. Important details were sometimes not handed over between professionals within and between primary and secondary care, in particular about women’s personal preferences. In case of referral of care from primary to secondary care, it was important for women that midwives handed over the care in person and stayed until they felt safe with the hospital team. Personal continuity of care, in which case the midwife stayed until the end of labour, was highly appreciated but not always expected.

Fear of transportion during or after labour was a reason for women to choose hospital birth but also to opt for home birth. Choice of place of birth emerged as a fluid concept; most women planned their place of birth during pregnancy and were aware that they would spend some time at home and possibly some time in hospital.

**Conclusions:**

In case of referral from primary to secondary care during labour, midwives should hand over their care in person and preferrably stay with women throughout labour. Planned place of birth should be regarded as a fluid concept rather than a dichotomous choice.

## Background

In several parts of the world, low risk women receive midwife-led care throughout childbirth [1]. When risk factors or complications occur, women in midwife-led care may be referred to obstetrician-led care for monitoring or medical interventions. In particular during labour, referral of care can greatly affect women’s childbirth experience. Rijnders showed that women who were referred during labour were more likely to look back negatively on their birth [2]. In another study, referred women were less satisfied with their birth experience and this was more pronounced for Dutch than for Belgian women [3]. However, it is not clear which aspects of care during referral contribute to a negative birth experience. A few qualitative studies have explored the experiences of women who were referred during labour in greater depth [4-6]. Rowe et al. interviewed women, who were transferred from a maternity unit to a consultant unit during labour, about their experiences. The maternity unit could be freestanding or alongside a consultant unit in hospital and women’s experiences of referral were therefore somewhat comparable to the Netherlands where women in primary care start labour either at home or in hospital in midwife-led care. In this study, discontinuity of care emerged as an important issue for women that affected their birth experience negatively [4].

Interestingly, one of the main aims of midwife-led models of care is to improve continuity of care by provision of care by one or a small group of midwives [7]. In some settings midwives stay with women after referral during labour and continue to provide care. In other setttings, for example in the Netherlands, midwives have no responsibility in women’s care anymore after referral and therefore often leave. The advantage of continuity in a midwife-led model of care then turns into a disadvantage of discontinuity of care during labour [4].

In the Netherlands, more than 80% of women start antenatal care in primary midwife led care and only 0.4% are looked after by general practitioners [8]. During pregnancy, one third of women are referred to obstetrician-led secondary or tertiary care because risk factors or complications occur [8]. About half of all pregnant women start labour in primary care and they can choose to give birth at home or in hospital assisted by their primary care giver. In hospital, they may give birth in an alongside midwifery unit or on the labour ward. If interventions such as continuous foetal monitoring, augmentation of labour or pain medication are required, women are referred to secondary care. In 2008, 57.4% of primiparous and 23.3% of multiparous women were referred during labour from primary to secondary care, mainly for non urgent reasons [9]. If women are on the labour ward already, they will stay in the same room and if they are in a midwifery unit or at home they will be moved to the labour ward. In case of an emergency at home, more than 80% of women are in hospital within 45 minutes from the moment a midwife called an ambulance [10]. After referral, primary care midwives have no medical responsibility anymore [11]. In many cases, care is taken over by a clinical midwife who works under the responsibility of an obstetrician [12].

Increasingly, it is recognised that the organisation of care in two separate echelons has many disadvantages, in particular in terms of discontinuity of care [11]. However, although most professionals are in favour of more integration between primary and secondary care to enhance continuity of care, there is no consensus on the ideal organisation of care (Perdok, *in press*). Before changes are made to the Dutch maternity care system, it is important to explore women’s views on important elements of care. Findings are important for other Western countries as well. The Dutch system has inspired changes in maternity care systems in other Western countries [13,14]. Changes to the Dutch system will therefore have an impact on these countries as well.

We conducted an interview study into the experiences of women who were referred during labour from primary to secondary care. In view of the importance of the issue of (dis)continuity of care when women are referred during labour, we decided to use this concept to focus our analysis. Haggerty et al. defined continuity of care as “the degree to which a series of discrete healthcare events is experienced as coherent and connected and consistent with the patient’s medical needs and personal context” [15]. They identified three important types of continuity of care, which were repeated by others in similar terms [16,17]. We defined these types as:

*Informational continuity*— All professionals involved in a woman’s intrapartum care have sufficient information about her medical situation and personal circumstances to be able to give appropriate care.

*Management continuity —* A consistent and coherent approach to management of a woman’s labour that is responsive to her changing needs.

*Relational continuity *— Continuity of care during labour provided by one health professional.

A unique aspect of referral during labour is that women may need to be transported from home to hospital. In Rowe et al.’s study, ‘the transfer journey’ emerged as an important theme for women that were transported from a freestanding midwifery unit to a hospital, which often caused uncertainty and anxiety. *Continuity of place* is therefore an additional, important type of continuity of care for women in labour, defined as ‘consistency of location during labour’.

In this study, we aimed to explore in-depth the experiences of women at term gestation who were referred during labour from primary to secondary care with regard to the four different types of continuity of care.

## Methods

### Design

We conducted a qualitative, semi-structured interview study into the experiences of women that were referred from primary to secondary care during labour in the Netherlands. The qualitative design enabled women to express in their own words how they experienced their labour. The ethical committee of VU University Medical Center confirmed that ethical approval was not necessary for this study (reference number 11/399). Interviews were conducted between three weeks and five months after birth, so that women would still remember details about their birth very well.

### Participants

Women included in the study were referred during labour from primary to secondary care at term gestation and, therefore, they would have been low risk at the onset of labour. We excluded women whose baby died or was seriously ill because we did not think it would be appropriate to ask them for an interview. Women who did not speak Dutch or English were also excluded. Purposive sampling was conducted to obtain a sample of women with variations in their planned place of birth, ethnic background, parity, education and place of residence [18]. In total, a convenience sample of 46 independent midwifery practices in rural and urban areas in different parts of the country were contacted by email or telephone to ask them if they were willing to recruit women for the study who started labour in primary care at term. Women that expressed an interest in the study were sent an information letter. Some women phoned the researchers if they were interested, others gave permission to their midwife to pass on their details. All women that were subsequently contacted by phone by one of the researchers (RS or IE) agreed to take part and they were interviewed between January and August 2012. After some time, midwives were asked to approach women with particular characteristics that were underrepresented such as non-Dutch ethnic background, multiparity and planned home birth. Recruitment stopped after data saturation had been achieved.

### Data collection

Women were interviewed in their own home by RS and IE. They were reassured that their names would be removed from the transcripts and that only the researchers would have access to the original interviews. They were encouraged not to give ‘desirable’ answers, for example about their caregivers, but to express their own opinions as this would help to improve the quality of maternity care. In particular, they were reassured their caregivers would not be informed about their views.

Women were asked to fill in a short questionnaire about their characteristics. For the interview, a topic list was used with the following subjects: detailed description of the birthing process, preparation for labour, care during labour, referral of care, cooperation between midwife and other professionals, postpartum care, current health, expectations for future birth and opinion on Dutch maternity care system. The questions were open ended to enable women to express their views freely in their own words. Slight adjustments were made to the topic list based on the first analyses to address important areas of interest in more depth. Interviews lasted 40 to 70 minutes, were audiotaped and transcribed verbatim and imported in Atlas-ti 5.2.

### Data analysis

Thematic analysis was conducted while data collection was ongoing, using the software program Atlas-ti, version 5.2, released 15-05-2006 [19]. Three researchers (AdJ, RS and IE) closely read all interviews. RS and IE coded two interviews separately from each other, using open coding. These codes were discussed by four researchers (RS, IE, MW, AdJ); RS and IE then coded all other interviews. Codes were subsequently clustered into sub-themes. Patterns in the data were identified and deviant cases were explored. The sub-themes were discussed in group meetings between the researchers. Most sub-themes were related to continuity of care. They included, for example, ‘expectations about the role of the midwife during labour’, ‘presence of the primary care midwife during and after referral’ and ‘transportation during labour’. Since discontinuity of care is a major issue when women are referred during labour, we decided to focus our analyses for this paper on the four aspects of continuity of care described earlier, through a process of theoretical thematic analysis [19]. According to Braun, theoretical thematic analysis tends ‘to be driven by the researcher’s theoretical or analytic interest in the area’ in contrast to inductive thematic analysis which is more data driven [19]. One researcher (AJ) filled in a matrix to summarise the data with the most important sub-themes concerning continuity of care in the column headings and respondent numbers in row headings. Examples of column headings were ‘involvement of client in decision-making’ ‘presence of midwife at the time of handover and at birth’ and ‘preferred place of birth and reasons why’. Finally, sub-themes were refined into final themes.

## Results

In total, 27 women were interviewed in Dutch who gave birth at term and who were in primary care at the onset of labour (Table 1). Of these, 9 women had a planned home birth of whom one made this decision at the onset of labour and the others during pregnancy. Of the 18 women with planned hospital birth, two started labour in an alongside midwifery unit. Two women were advised by their midwives to give birth in hospital.

**Table 1:** Characteristics of the 27 women included in the study

**Respondent**	**Ethnicity**	**Education**	**Place of residence**	**Parity**	**Women’s reported reason for referral to secondary care**	**Mode of transport to hospital**	**Planned place of birth**	**Intended planned place of birth next time**
**R1**	Dutch	Academic	City	1	Request for pain medication	Car	Hospital	Hospital
**R2**	Dutch	Higher vocational	City	1	Request for pain medication	Car	Hospital	M.i.
**R3**	Dutch	Academic	City	3	Failure to progress first stage	Car	Hospital	Hospital
**R4**	Dutch	Higher vocational	City	1	Failure to progress first stage	Car	Hospital before labour, considered home at onset of labour	Hospital
**R5**	Dutch	Medium vocational	Village	1	Failure to progress second stage	Ambulance	Home	M.i.
**R6**	Dutch	Academic	City	1	Meconium stained liquor	Car	Hospital	Hospital, but wants to stay longer at home
**R7**	Dutch	Academic	City	1	Request for pain medication, did not receive it, then referred for failure to progress in second stage	Car	Alongside midwifery unit	M.i.
**R8**	Polish	Higher vocational	City	1	Prolonged ruptured membranes	Car	Hospital but stay at home as long as possible	Undecided
**R9**	Dutch	Higher vocational	City	1	Failure to progress first stage	Car	Undecided before labour, during labour home	M.i.
**R10**	Dutch	Higher vocational	Village	1	Foetal distress second stage	Ambulance	Home	Home
**R11**	Surinamese	Medium vocational	City	1	Failure to progress first stage, received pain medication immediately after tramsfer of care	Car	Hospital because of high BMI, preferred home	Hospital because of high BMI
**R12**	Dutch	Higher vocational	City	1	Failure to progress second stage	Ambulance	Home	Hospital
**R13**	Dutch	Higher vocational	Village	1	Meconium stained liquor	Car	Hospital	M.i.
**R14**	Dutch	Secondary school	Village	2	Failure to progress first stage	Car	Hospital	Hospital
**R15**	Dutch	Academic	City	1	Failure to progress second stage	Car	Hospital	M.i.
**R16**	Surinamese	Higher vocational	City	1	Meconium stained liquor	Car	Hospital	Hospital
**R17**	Dutch	Medium vocational	Village	1	Request for pain medication	Car	Hospital	M.i.
**R18**	Dutch	Higher vocational	City	1	Request for pain medication	Car	Hospital	Hospital
**R19**	Dutch	Higher vocational	City	1	Request for pain medication	Car	Hospital	M.i.
**R20**	Dutch	Higher vocational	City	1	Request for pain medication, did not receive it, then referred for meconium stained liquor and foetal distress	Car	Home	Hospital
**R22**	Dutch	Higher vocational	City	2	Meconium stained liquor	Car	Home	Home
**R23**	Polish	Academic	City	2	Request for pain medication, did not receive it	Car	Alongside midwifery unit	Alongside midwifery unit
**R24**	Dutch	Medium vocational	Village	2	Meconium stained liquor	Car	Hospital because of previous postpartum haemorrhage	M.i.
**R25**	Dutch	Medium vocational	Village	1	Failure to progress second stage	Ambulance	Home	Home
**R26**	Dutch	Medium vocational	Village	1	Request for pain medication	Car	Hospital	M.i.
**R27**	Dutch	Medium vocational	City	3	Request for pain medication	Car	Home	Home
**R28**	Dutch	Medium vocational	City	2	Meconium stained liquor	Car	Home	Home

M.i. = medical indication for hospital birth, such as previous caesarean section or previous postpartum haemorrhage. R21 was excluded because this woman gave birth preterm.

The most important themes that emerged during the analysis with regard to the four aspects of continuity of care are summarised below, illustrated by quotes that were translated into English and carefully edited slightly to make them more readable without loss of meaning. We identified the following six themes: 1. Where professionals cooperate, information may get lost (informational continuity), 2. Involvement in decision making (management continuity), 3. A consistent and coherent approach to labour management (management continuity), 4. Management continuity is silver, relational continuity is gold (relational continuity), 5. The role of transportation in planned place of birth (continuity of place) and 6. Planned place of birth as a fluid concept (continuity of place).

### Informational continuity; where professionals cooperate, information may get lost

It was important for women that professionals were aware of their medical situation and about their personal preferences. Although women made many positive comments about the cooperation between professionals, their remarks showed that information gets lost sometimes during handover of care between caregivers within and between primary and secondary care.

One woman had the impression that her primary care midwife had not handed over to her colleague at the end of her shift that she wanted pain medication. Three women thought that staff in hospital was very busy and therefore information was not handed over properly between them.

After referral of care women were looked after by secondary care professionals who they usually never met before. Nevertheless, many women thought that professionals in secondary care had received enough information about them. Some commented that they carried their birth plan or pregnancy notes which helped to inform secondary care professionals about their circumstances and preferences.

On the other hand, several women said that information about them got lost during handover from primary to secondary care. In particular, non medical information, for example on women’s preferences, was not always handed over:

R2: Yes, because every consultation at the midwife […] something is written down again. While now in retrospect I think, there was no point, because it didn’t help me at all during labour. During labour you have to give all that information again, [laughs..] (primiparous woman referred for pain medication)

### Management continuity; involvement in decision-making

Women thought it was important that they had been sufficiently prepared about the options and logistics during labour in the Dutch maternity care system. This would help them to be involved in decision-making before and during labour.

Women varied in the amount of information they wanted to receive on the possibility of referral and on potential complications. Most women said they received sufficient information. A few women received more information than they wanted to hear. Some women mentioned that they were informed about the chance of referral and medical interventions but did not apply this information to themselves and anticipated that they would not need to be referred. Some thought that their primary care midwives did not give them sufficient information. One woman did not feel prepared for the possibility of needing a caesarean section:

R19: Before I was pregnant, I never looked for information about caesarean sections […]. And now in retrospect I think, yes, at such a primary care midwifery practice … you are there nine months of course and then it is really very important that women much more… that they are also prepared for that. […] because it is such a blow, really such a blow…(primiparous woman, referred for pain medication).

Most women felt they were involved in making important decisions and were taken seriously when they asked for pain medication. This did not mean they always received pain medication because sometimes labour progressed fast and women understood and accepted that they did not receive it. For most women it appeared more important that they felt cared for and involved in decision-making than whether they got pain medication or not:

I: Are there things that you would have liked differently during your referral?

R7: No, no, but I think that’s because X [the midwife] remained so involved… she stayed (primiparous woman, requested pain medication but did not get it because her cervix was almost fully dilated, later on she was referred for failure to progress in second stage)

In contrast, another primiparous woman who did not receive pain medication, because labour had progressed too far, was unhappy about this. She feels in retrospect that the Dutch society is too much in favour of home birth and that women are made to believe that they can give birth without pain medication. She would go to hospital earlier next time in order to get pain medication. Two other women commented that maternity care professionals should be less reluctant to give pain medication or perform medical interventions.

### Management continuity; a consistent and coherent approach to labour management

Women expected midwives to refer care to hospital staff in person and to stay with them until they were settled and trusted the hospital staff. This made them feel safe.

All but two women were accompanied by their primary care midwife to hospital. Two women were not visited by their midwife but referred via telephone. One of them needed pain medication. Her midwife had seen her in early labour but was busy with another labouring woman when she phoned again. She implied that she would have felt more secure if the midwife had handed over in person:

R2: Well, it might have been nice for myself if the midwife would still come to hospital. […] So that you know, the midwife has contact with somebody from the hospital now.. and they do your handover (primiparous woman)

One woman had gone to hospital without the midwife during her previous labour. This time the midwife came with her but had to leave before the actual birth to another labouring woman. Nevertheless, her experience was much better because she already got used to the hospital staff when the midwife left.

The majority of women said the handover from primary to secondary care went smoothly, even though they were not always aware of how information was exchanged between professionals. Several of them mentioned that they experienced consistency in the way care was delivered:

R14: The [primary care] midwife said like: if you want something you just have to make it known and then you just have to ask… And the obstetrician did that too […]. That was like…. as if the same person had talked to me the whole time. Because it all continued in a similar manner….(multiparous woman, referred for failure to progress during first stage).

### Management continuity is silver, relational continuity is gold

The primary care midwives of eight of the women stayed after referral until the baby was born, even though they did not have any medical responsibility anymore. Women appreciated this very much:

I: How would your experience have been if she had left?

R16: I would have regretted it very much but that little one needs to get out so at that moment I would go along with whatever happened….. But I would have felt much less at ease I think.(primiparous woman, referred for meconium stained liquor).

Women sometimes felt more confident about the need for medical interventions if their primary care midwife stayed and could explain this to them. On the other hand, the midwife could also act as an advocate and point out women’s preferences to hospital staff which could lead to flexibility in the hospital routines:

R10: …the obstetrician came to say, we actually want to put up a drip. And then she has, the midwife has spoken for me and said, we would like things to be as natural as possible […] Then they did not start again about the drip, that was just finished. (primiparous woman, referred for suspected foetal distress during second stage).

Nineteen women gave birth after their primary care midwife left. Many women said they would have liked their midwife to stay but they understood and accepted that this was not always possible. A few women commented that it is strange that you build up a rapport with primary care midwives over the course of nine months and then have to get used to new professionals after referral during labour.

On the other hand, two women thought it is good to have a division between primary and secondary care, because ‘*the hospital does not come to your house’*. They appreciated very much that the primary care midwife came to their house, enabled them to stay at home as long as they wanted or was medically possible and accompanied them to hospital.

A few women who did not know their primary care midwife very well did not mind whether she stayed or not. Some women mentioned that it was more important to them that health professionals were competent and that they felt safe than that they knew them. If women had developed a good rapport with an obstetric nurse or clinical midwife, this seemed to compensate for the absence of the primary care midwife.

### Continuity of place; the role of transportation in choice of place of birth

When planning their place of birth, the need for transportation was an important factor to consider for women. Four women who planned a hospital birth mentioned that they did not want to risk being transported in an emergency and two of them were worried about having to be lifted out of the house by the fire brigade, because they did not live on the ground floor. Nine women chose a hospital birth to be close to medical assistance if need be:

R6: I want to be in the right place if something is not right. You hear all kind of stories from people who had to go to hospital at the last minute and lots of panic…(primiparous woman, referred for meconium stained liquor)

On the other hand, six of the nine women who planned to give birth at home also mentioned the avoidance of transportation during or after childbirth as an important reason for their choice. Even though all women had to be transported to hospital eventually, most did not regret that they tried home birth. One multiparous woman was referred during her first labour and this time had to be transported by ambulance:

R28: yes that’s why, yes in nine out of ten cases you start labour at home of course…and yes the advantage for me was, such a trip in the ambulance was not pleasant, but to get into a car in such a state, yes, then I felt it was very pleasant to go by ambulance, than you lie down a bit more comfortably than when you are curled up in a car, say…

### Continuity of place; planned place of birth as a fluid concept

Although all women had a preference for a certain place of birth, many talked about part of the labour being at home and part of it in hospital. Women with planned hospital birth varied in the amount of time they would like to labour at home first. All women with planned home birth were transported to hospital during labour, but many did not talk about their experience as a hospital birth, but rather as a home birth with some assistance from the hospital at the time they needed it. A multiparous woman who was referred for meconium stained liquor:

R28: I have had both sides of course, eh… I did a large part at home and only the last bit in hospital and that was a very good experience.

Five women with planned home birth would make the same choice again. Some felt that the time they spent at home may have contributed to a better outcome.

R25:… I would try home again next time. It is not that I now think… immediately hospital next time, no, I think like… perhaps the journey to hospital was just necessary. Had I been in hospital straight away, maybe I would have needed a vacuum pump.. you just don’t know…no, I wouldn’t have liked it any other way, no. (primiparous woman, referred for failure to progress in second stage)

Two women with planned home birth have a medical indication for hospital birth next time. The woman who was referred from home for pain medication but who did not receive it because her cervix was almost fully dilated was the only one who regretted having tried home birth. She said the thought of having to get into a car made her stay at home longer. One other woman did not regret choosing home birth but would like to give birth in hospital next time and described the ambulance journey as ‘hell’.

Of the eighteen women who planned hospital birth, nine have a medical indication to give birth in hospital next time. Of the other women, one did not yet make up her mind where she would like to give birth next time. The other eight women would choose hospital birth again next time. Two of them mentioned that they would like to stay at home longer next time before going to hospital. On the other hand, some women would like to go to hospital earlier although some expressed doubts whether they would be ‘allowed’ to go to hospital in early labour by their midwife. Some women tried to balance between staying at home as long as possible to enjoy the comfort of their own environment versus going to hospital before they were in advanced labour.

R4: eh … well I thought it was top, but what I would like differently next time is that I want to go to hospital much earlier next time. […] Yes, but yes, on the other hand, where do you stay then, eh..? Look, here you have a very nice little room and bed and eh, you are in your own environment. But where would you then stay all those hours, I don’t know…(primiparous woman, referred for failure to progress in first stage).

## Discussion

### Principal findings

Continuity of care was very important for women who were referred during labour because this contributed to their feeling of safety. Although many women commented that professionals had enough information about them, important details were sometimes not handed over between professionals within primary and secondary care and between the levels of care, in particular about women’s personal preferences.

A personal handover between primary and secondary care was seen as essential by women for management continuity of care. This implied that the midwife handed over a woman’s care in person and stayed until the woman was settled and felt safe with the hospital team. Personal continuity of care, in which case the midwife stayed until the end of labour, was highly appreciated but not always expected.

Fear of transportation during or after labour was a reason for women to choose hospital birth but also to opt for home birth. Choice of place of birth emerged as a fluid concept; most women planned their place of birth during pregnancy but were aware that they would spend some time at home and possibly some time in hospital depending on women’s preferences and how labour progressed.

### Strengths and limitations

A strength of our study is that it was conducted in a country where choice of place of birth for low-risk women is still a structural element of the maternity care system. The findings may be relevant to countries that are increasingly giving women choices to have midwife-led care and home birth. The study was conducted by a multidisciplinary team of researchers. They regularly met and discussed the ongoing analyses to enhance validity of the results.

Our study has some limitations. We only included women who spoke Dutch and therefore could not interview certain ethnic minority women. In addition, midwives approached women to ask them if they were willing to take part in the study. In theory, they might have been selective and not asked women who were dissatisfied with their care. However, many women expressed critical comments and therefore this does not appear to have been a major problem.

### Implications

The importance of continuity of care for women in order to feel safe during labour is consistent with findings in other studies [5,20-23]. It is important that professionals handover medical information and information on women’s preferences about labour to each other. Women indicated that carrying their notes or a birth plan helped in the transfer of information if they were referred from primary to secondary care. In a meta-synthesis of qualitative studies into patients’ perceptions of continuity of care, patients also considered health records to be important in maintaining informational continuity [17]. In addition, they valued information technology that is accessible at any point of care. An American study showed that on the labour ward more vital clinical information was missing when paper records were used compared to electronic health records [24]. Hence, modern information technology is not only important to facilitate communication between levels of care but also between professionals within primary or within secondary care.

In the Netherlands, midwives often do not stay with the woman after referral and sometimes do not accompany her to hospital if she is transferred from home. Women in our study made it clear that they expected midwives to accompany them. Similarly, in Rowe et al’s study women pointed out that it was important to them that midwives personally handed over the care to another midwife [4].

Many women in our study did not expect midwives to stay until the birth but appreciated it very much if they did. A literature review showed that being cared for by the same person throughout labour is important to women and far more relevant than having met the caregiver during pregnancy [21]. In some countries, such as Canada, midwives will always continue to provide support if women need consultant care and this contributes to their positive experience about planned home birth [25]. In Canada, primary care midwives have a broader scope of practice which means they continue to provide care to women who need medical interventions for moderate risk indications, such as meconium stained liquor [26]. Therefore, the number of women that are referred to obstetrician-led care is considerably lower than in the Netherlands [27]. This enhances personal continuity of care during labour. Currently, expansion of the scope of practice of primary care midwives is considered in the Netherlands and will be evaluated in some pilot projects. Our results suggest that these initiatives may help to meet women’s preferences.

A surprising finding in the study was the fact that most women with planned home birth did not regret their choice and most would choose home birth again next time. Although one woman described the ambulance journey as ‘hell’, others mentioned positive aspects of the journey as well. This contrasts the study of Rowe et al. [4]. They described that all women who planned birth in a freestanding maternity unit were transported by ambulance and for most the journey was very unsettling. In our study, only four of the nine women with planned home birth were transported by ambulance, the others travelled with their own car. Other factors that may explain the differences in findings may be that travel distances are often short in the Netherlands [28] and, unlike in the United Kingdom, planned home birth has always been a well established aspect of the maternity care system. Women may therefore be more familiar with the possibility of referral.

Although transportation was an important factor in the choice for place of birth, this did not only apply to emergency transport but equally to transportation by car during or after labour. This is consistent with findings of a Dutch study [29]. Women who gave birth in hospital complained that they had to get into their car shortly after they gave birth and women who planned home birth mentioned the fact that medical help was not nearby as a disadvantage. In a Canadian study too, the thought of having to transport to hospital in established labour made some women choose home birth [30].

Our findings suggest that choosing place of birth is a fluid concept rather than a dichotomous choice. Although other studies too have shown that women make their choice of place of birth in the knowledge that labour is unpredictable and therefore their expectations may not come true [25,30], the discourse on place of birth focuses on a dichotomy between planned home or planned birth centre versus planned hospital birth [25,27,30-33]. There are several reasons to address planning place of birth as a fluid concept.

Firstly, women described their labour as partly at home, partly in hospital, regardless of where they gave birth. Virtually all spontaneous labours start at home. Women vary in the amount of time they want to spend at home if they plan hospital birth, but also in their willingness to persevere without pain medication if they plan home birth.

Secondly, a large number of women do not give birth in the place they intended. More than 30% of all women that start labour in primary care in the Netherlands are referred to secondary care [34]. On the other hand, in one Dutch study, 9% of primiparous women and 25% of multiparous women who planned hospital birth gave birth at home [35]. These women may have laboured too fast to go to hospital or they may have changed their mind during labour. All women in primary care are visited by their midwife at the beginning of labour, regardless of their planned place of birth. At some point during an uneventful labour, the woman will need to decide whether to stay at home or to go to a birth centre or to hospital. If she wants to give birth at home, the midwife will call the maternity care assistant. Otherwise, she will accompany the woman to a birth centre or to hospital. It is essential that women are prepared for the different scenario’s that can occur during labour [4].

Regarding planned place of birth as a fluid concept will influence the process of decision-making around place of birth. Rather than presenting place of birth as a dichotomous option, women should be informed about advantages and disadvantages of home, birth centre and hospital during the various stages of labour. This information should include information on the benefits of labouring in a familiar environment versus the possibility of medical interventions, including pain medication in hospital. Information should also be given on advantages and disadvantages of transportation to and from hospital at different stages of labour and on the unpredictability of the labour process. Based on their preferences women make a tentative plan for the process of labour and the actual place of birth, knowing that the plan can be revised at different points during labour, based on the medical circumstances and women’s experiences at the time. Part of this plan is that women are aware that they will spend some time at home if they go into spontaneous labour. Additionally, they may spend some time in a birth centre or in hospital if they wish to or if medical circumstances make it necessary. Such a ‘process’ approach may make women feel less disappointed that they were ‘not doing as well’ if they did not give birth in the place they planned [4].

## Conclusions

Continuity of care was very important for women who were referred during labour because this contributes to their feeling of safety. In case of referral from primary to secondary care during labour, it appears to be essential for women that midwives hand over in person and stay until they feel safe with the hospital team. Personal continuity of care by one professional throughout labour is highly appreciated by many women. Fear of transportion during or after labour is a reason for women to choose hospital birth but also to opt for home birth. Planned place of birth should be regarded as a fluid concept rather than a dichotomous choice and includes planned time spent at home and time spent in a birth centre and in hospital depending on women’s preferences and the labour process.

## Competing interests

“All authors have completed the Unified Competing Interest form at http://www.icmje.org/downloads/coi_disclosure (available on request from the corresponding author) and declare: no support from any organisation for the submitted work; no financial relationships with any organisations that might have an interest in the submitted work in the previous 3 years; no other relationships or activities that could appear to have influenced the submitted work”. The authors declare that they have no competing interests.

## Authors’ contributions

AJ conceived the idea for the study, wrote the article and is guarantor of the study. RS and IE conducted the interviews, coded the transcripts. All authors contributed to interpretation of the data, they critically revised earlier drafts of the paper for important intellectual content and gave final approval of the version to be published. The researchers had access to all the research data.

## Pre-publication history

The pre-publication history for this paper can be accessed here:

http://www.biomedcentral.com/1471-2393/14/103/prepub
